# Microvessel Chaste: An Open Library for Spatial Modeling of Vascularized Tissues

**DOI:** 10.1016/j.bpj.2017.03.036

**Published:** 2017-05-09

**Authors:** James A. Grogan, Anthony J. Connor, Bostjan Markelc, Ruth J. Muschel, Philip K. Maini, Helen M. Byrne, Joe M. Pitt-Francis

**Affiliations:** 1 Wolfson Centre for Mathematical Biology, Mathematical Institute, University of Oxford, Oxford, United Kingdom; 2 Department of Computer Science, University of Oxford, Oxford, United Kingdom; 3 CRUK/MRC Oxford Institute for Radiation Oncology, University of Oxford, Oxford, United Kingdom

## Abstract

Spatial models of vascularized tissues are widely used in computational physiology. We introduce a software library for composing multiscale, multiphysics models for applications including tumor growth, angiogenesis, osteogenesis, coronary perfusion, and oxygen delivery. Composition of such models is time consuming, with many researchers writing custom software. Recent advances in imaging have produced detailed three-dimensional (3D) datasets of vascularized tissues at the scale of individual cells. To fully exploit such data there is an increasing need for software that allows user-friendly composition of efficient, 3D models of vascularized tissues, and comparison of predictions with in vivo or in vitro experiments and alternative computational formulations. Microvessel Chaste can be used to build simulations of vessel growth and adaptation in response to mechanical and chemical stimuli; intra- and extravascular transport of nutrients, growth factors and drugs; and cell proliferation in complex 3D geometries. In addition, it can be used to develop custom software for integrating modeling with experimental data processing workflows, facilitated by a comprehensive Python interface to solvers implemented in C++. This article links to two reproducible example problems, showing how the library can be used to build simulations of tumor growth and angiogenesis with realistic vessel networks.

## Introduction

Spatial models of vascularized tissue are used to study the growth and response to treatment of tumors (1), angiogenesis (2), osteogenesis (3), coronary perfusion (4), and tissue oxygenation (5). Such models typically comprise a combination of the following: 1) agent-based or continuum representations of migrating and proliferating cells; 2) line-based or spatially resolved representations of microvessels; 3) the solution of blood, nutrient, growth factor, and drug transport problems in vessel networks whose geometry and connectivity may evolve; 4) the solution of growth factor and drug transport problems in the evolving extravascular space; and 5) vessel formation and endothelial tip cell migration in response to mechanical and chemical cues.

Composition of spatial models of vascularized tissues is a time-consuming process, with most researchers writing custom software. Examples of such multiscale/hybrid models include those developed by Anderson and Chaplain (6), Alarcón et al. (7), Frieboes et al. (8), Shirinifard et al. (9), Owen et al. (1), Perfahl et al. (10), Welter and Rieger (11), Secomb et al. (2), and Boas and Merks (12). The need to account for many biological phenomena, including multiscale spatial processes occurring over different timescales, has made the development of more general software frameworks challenging (13, 14). Tools for integration with experimental imaging data and model benchmarking and cross comparison are also important (14, 15). Several groups, including Liu et al. (16), Secomb et al. (2), and Beard et al. (17), have produced more general software that focuses on modeling and integration with imaging data. Notable efforts in the development of vascularized tissue models have been undertaken as part of the broader European Union’s Virtual Physiological Human Project (http://www.vph-institute.org/) and the National Institute of General Medical Sciences’ Virtual Physiological Rat (http://www.virtualrat.org/) program.

In this article, we introduce Microvessel Chaste, an open-source Python/C++ library for composing spatial models of vascularized tissues. It is designed so that users can assemble their own models from a collection of building blocks, based on established numerical libraries. It is a plug-in for the Chaste C++ library for problems in computational physiology and biology (18), adding functionality for modeling microvessels and complex, evolving three-dimensional (3D) tissue domains. Development has been motivated by the above-mentioned models and software, following similar goals in the development of detailed models of vascularized tissues and integration of experimental data. However, there is an additional focus on providing a user-friendly, general framework for model composition, in a manner similar to that in which Chaste (18), CompuCell3D (19), EPISIM (20), and PhysiCell (21) can be used to compose tissue models with agent-based representations of cells. A novel (to our knowledge) and important feature of Microvessel Chaste is its comprehensive Python interface, which facilitates integration with a growing collection of scientific Python software for image processing, statistical analysis, and visualization. The library has facilitated the integration of modeling with high-resolution 3D imaging data, as shown in Grogan et al. (22) and below, and will be useful in future model cross-comparison studies.

The remainder of the article is structured as follows. The next section introduces the main components of the Chaste and Microvessel Chaste libraries. We then present examples showing how these components can be combined to generate numerical solutions of biophysical models of interest, focusing on tumor growth and angiogenesis. Integration with experimental imaging data is also demonstrated.

## Materials and Methods

**Figure 1:**
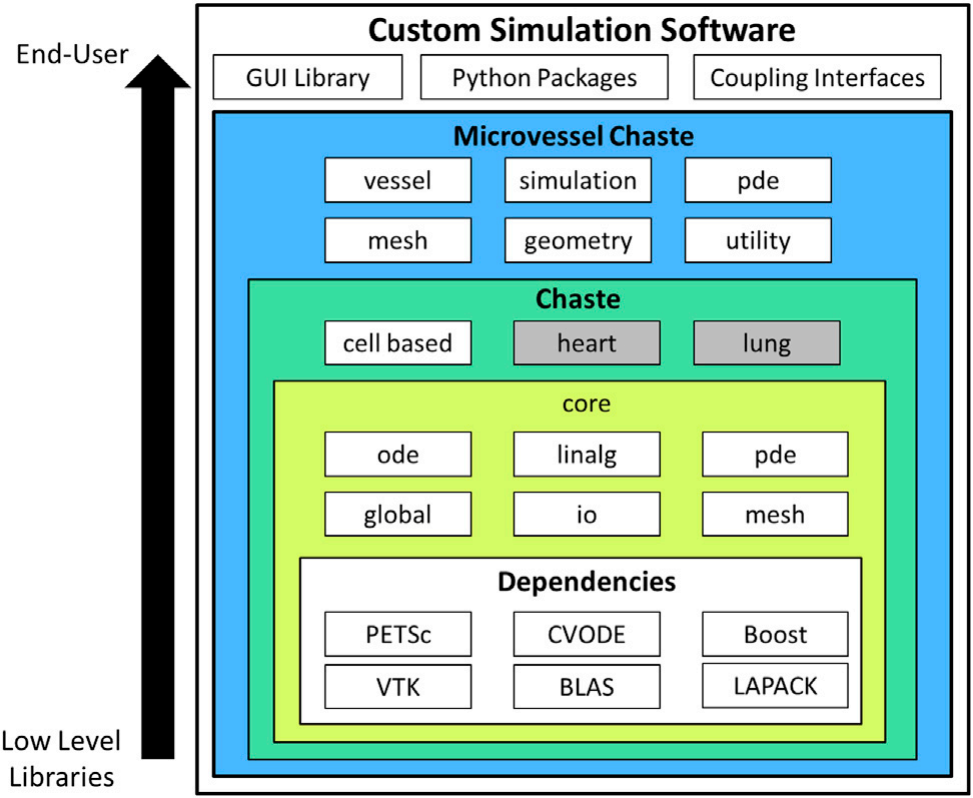
Given here is a schematic showing the components used in the Microvessel Chaste library, and how they can be used to generate custom simulation software. Shaded components are not currently used in the library. To see this figure in color, go online.

Fig. 1 shows the main components of Chaste and Microvessel Chaste and how they build on well-known numerical libraries. Mirams et al. (18) and Osborne et al. (15) should be consulted for a detailed description of the Chaste library and its use in individual cell-based models, respectively. However, a brief summary is given here. These components can be used to assemble custom models for a broad variety of vascularized tissue applications in a flexible and modular way.

Chaste is a collection of C++ classes, interfacing established parallel linear algebra, ordinary differential equation (ODE) solver, and visualization libraries. The core component has low-level functionality for solving ODE systems, constructing finite-element solvers, reading and writing meshes, and general linear algebra operations. Interfaces to underlying libraries provide full access to solution controls, such as time stepping and tolerances. The component also has a selection of prebuilt finite-element solvers for elliptic and parabolic partial differential equations (PDEs), coupled ODE and PDE systems, and nonlinear mechanics problems, allowing users to construct detailed and bespoke biophysical tissue models (18). The core functionality is extended by the cell-based component, allowing modeling of tissues with agent-based descriptions of cells. Functionality includes cell cycle modeling, based on the solution of ODE systems, and intra- and extracellular chemical transport modeling, based on the solution of PDEs with cells acting as discrete sinks or sources of the chemicals. Various discrete on- and off-lattice cell representations and mechanical models are available, including cellular Potts, vertex-based methods, and center-based methods (15).

The Microvessel Chaste library uses the Chaste components through their C++ API. It adds functionality for modeling microvessels using line- or surface-based descriptions, allowing a wide range of existing models to be implemented or extended (1, 10, 22). The geometry component is a collection of tools for construction and manipulation of 3D volumes and surfaces, which is useful for detailed modeling of microvessel walls or anatomical features such as the cornea, demonstrated below. The vessel component has tools for artificial vessel network construction, reading real networks from file, and network characterization (such as the construction of line or branch density maps). The simulation component contains many well-known submodels of vessel network blood flow, including nonlinear blood rheology (23), plasma splitting (24), structural adaptation in response the chemical and mechanical cues (25), and sprouting angiogenesis (1). The Microvessel Chaste library also extends existing Chaste components. The extended PDE component contains PETSc-based finite difference solvers, allowing chemical transport PDEs to be solved in a manner typical of the literature (1). Chemical release and uptake from vessels can be modeled by including their action as discrete sink or source terms in chemical transport PDEs (1). The mesh component has tools for automatic meshing of complex 3D geometries, facilitating construction of detailed models of growing tissues.

The reader is referred to Owen et al. (1), Perfahl et al. (10), and Grogan et al. (22) for detailed descriptions of the biological background of incorporated submodels, how they can be coupled, and how they are parameterized using experimental data. Because Microvessel Chaste is a library, users can build their own models, choosing suitable submodels, coupling schemes, and time-stepping strategies. The aforementioned publications (1, 10, 22), web-based tutorials, and software API documentation provide guidance in this regard. Most default experimental parameters in the software are tagged with a literature source and typed over unit, while API documentation and solver names indicate the original literature sources for submodels.

## Results and Discussion

In this section, tumor growth and angiogenesis problems are demonstrated; they are available for reproduction at https://jmsgrogan.github.io/MicrovesselChaste. A collection of additional, simpler examples is also available at this location. The examples are simplified to facilitate the tutorial format and reproduction by the reader. Careful parameterization is required before they can be used to gain new biological insights. The examples cover only a small selection of available functionality, and readers are encouraged to consult the software’s web page and API documentation for further details.

### A 3D tumor growth simulation

**Figure 2:**
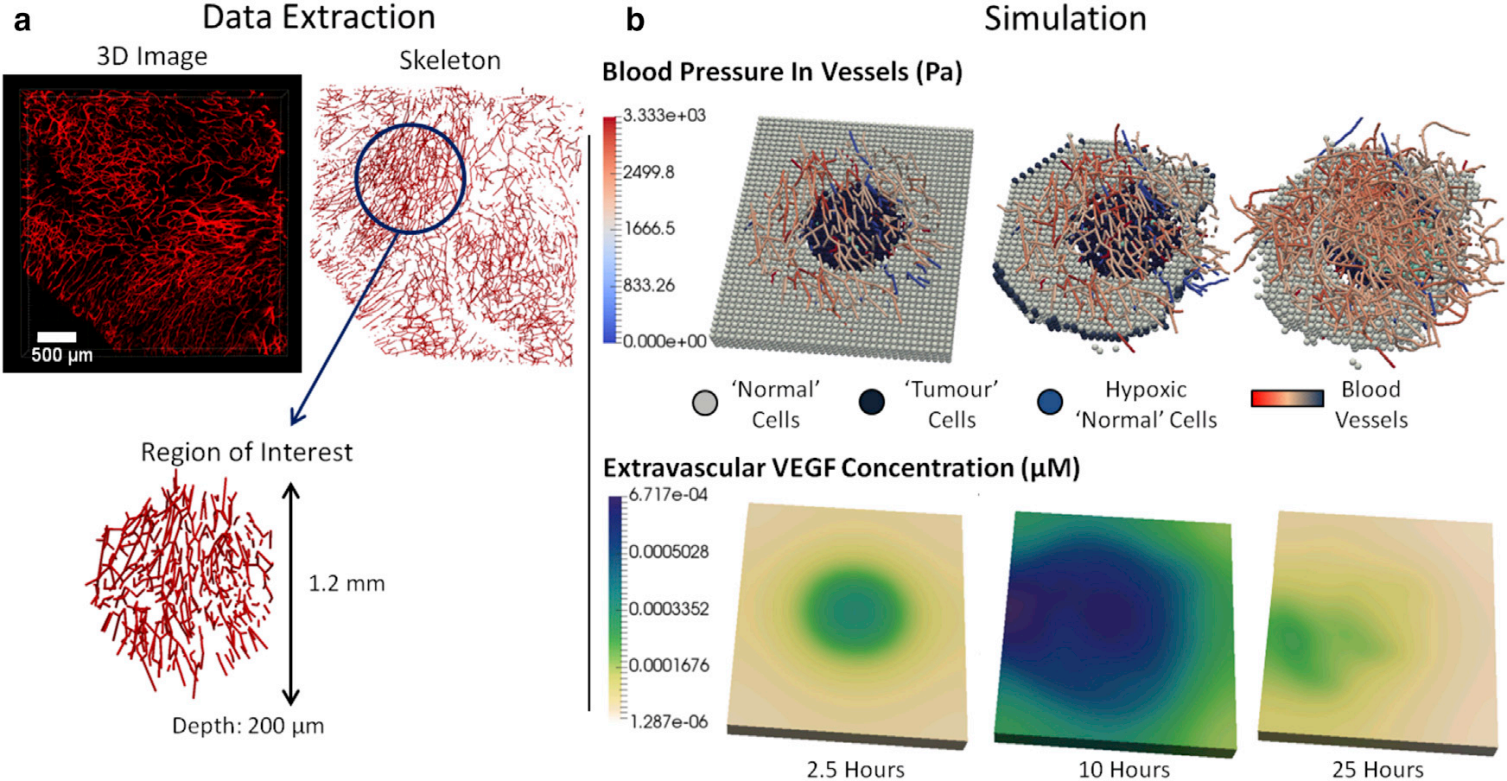
(*a*) A 3D intravital image of a tumor microvessel network (*red*) is obtained and a skeleton extracted as described in Grogan et al. (22). A cylindrical region of interest with diameter 1.2 mm is extracted for the example simulation. (*b*) A tumor growth simulation using the extracted microvessel network and Microvessel Chaste is given. The predicted evolution of the tumor over 25 h is shown, including blood pressure in growing vessels, VEGF concentrations in the extravascular space, and discrete cells. To see this figure in color, go online.

The first example, shown in Fig. 2, is a 3D simulation of tumor growth in a vessel network geometry obtained using multiphoton imaging after implantation of MC38 tumor cells in a mouse (22). This hybrid, multiscale tumor growth model is similar to many in the literature (6, 8, 10, 26), and in particular, uses submodels and parameter values described in Owen et al. (1). However, the use of a large, realistic and evolving tumor vessel network distinguishes this example from previous studies. The simulation is facilitated by recent advances in intravital imaging, which allow in vivo observation of tumor growth at the scale of individual cells, and the new preprocessing and modeling functionality in Microvessel Chaste. The vessel network preprocessing time was 6 s, and 25 simulated hours of tumor growth took 25 min on a standard desktop PC.

The problem is initialized with a 3D region of the tumor vessel network. A regular lattice with spacing of 40 *μ*m is generated in the bounding box of the network geometry, using the Microvessel Chaste mesh and vessel components shown in Fig. 1. A cellular automaton-based cell population fills all lattice sites, including those occupied by vessels, using the Chaste cell-based component. “Tumor” cell types are assigned to a 300-*μ*m-diameter central cylindrical region and “Normal” types to the remainder. Cell cycling is represented by a subcellular model described in Owen et al. (1), which leads to oxygen-dependent proliferation rates and vascular endothelial growth factor (VEGF) release rates that differ across cell populations. The cycle model is solved as a system of ODEs using the Chaste ODE component, with a time step of 5 min. Cells far from oxygen-rich vessels experience low oxygen levels and, as a result, become hypoxic and release VEGF. Oxygen and VEGF transport in the domain are each described using a typical steady-state reaction diffusion PDE of the following form (1):

(1) $$D\nabla^{2}c+\rho \left(c_{b}-c\right)+kc-\lambda c=0,$$

where *c* is the extravascular concentration of oxygen or VEGF; *D* is an effective diffusivity; *c*_*b*_ is the vascular concentration; *ρ* is an effective vascular permeability that is nonzero and positive only on lattice sites occupied by vessels; *k* is a cell consumption or release rate, depending on species, and is nonzero only on lattice sites occupied by cells; and *λ* is a positive rate of natural decay, only relevant for VEGF. PDEs are solved on the same regular grid as the cellular automaton, using the finite difference solver in the Microvessel Chaste PDE component. Many alternative PDEs can be constructed using the software, including the addition of general nonlinear sink and source terms and relaxation of the steady-state assumption. The solver is constructed in a standard way using available PETSc features. Transport PDEs are solved and cell positions are updated at each 30-min global time step. Global time stepping is managed by the Chaste cell-based component. VEGF stimulates the sprouting and chemotactic migration of new vessels from the existing vasculature. Sprouts form at a rate dependent on VEGF concentration and perform a lattice-free persistent random walk biased toward nearby vessels and positive VEGF gradients, using functionality in the simulation component. Off- and on-lattice tip migration submodels are easily switched, allowing model comparisons. Vessel movement occurs once per global time increment. Further details on modeling, coupling, and time stepping can be found in Owen et al. (1).

Blood flow is modeled in the branching vessel network using a typical 1D simplification (23). A pressure difference is applied across the network with a drop of 1.33 kPa through the 200-*μ*m depth. Only perfused vessels deliver oxygen. Vessel radii can change in response to mechanical stimuli following typical models in the literature (25). Blood flow and structural adaptation models were used from the simulation component. At later times, cells far from the vessels become apoptotic. The surviving tumor cells gradually invade the domain at the expense of the normal cells. This process is similar to those observed in the simulations of Perfahl et al. (10), Anderson and Chaplain (6), and others. There are many potential extensions to models of this type, including simulated administration of chemotherapeutic and antiangiogenic drugs (7) and radiotherapy (22). These cases can be simulated using the Microvessel Chaste library.

### A 3D angiogenesis simulation in a curved domain

**Figure 3:**
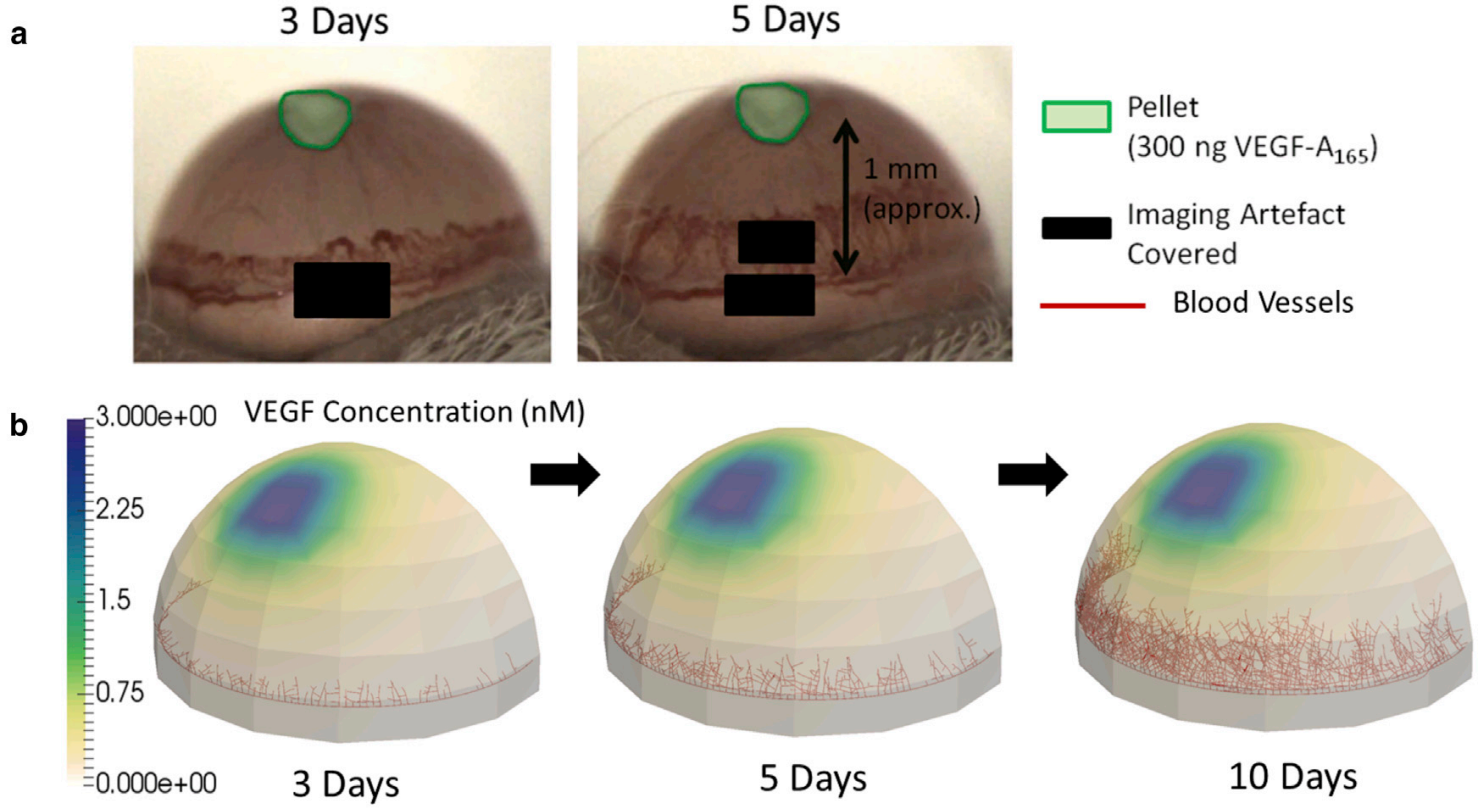
(*a*) Given here are images from a cornea micropocket experiment showing microvessels (*dark red*) at 3–5 days postpellet implantation (26). (*b*) Shown here is application of the Microvessel Chaste library in modeling a similar experiment. To see this figure in color, go online.

Our second example is a 3D, off-lattice simulation of angiogenesis in a curved geometry (see Fig. 3). This example demonstrates geometry manipulation and the solution of PDEs on 3D domains. Again, the example is simplified for the purposes of a tutorial and requires careful parameterization before it can be used to gain biological insights. The application is appropriate for the corneal micropocket assay that is widely used to study angiogenesis (26). Typical experimental results are shown in Fig. 3 *a*. The simulation time was 10 s on a standard desktop PC.

In this experimental assay a pellet containing an angiogenic growth factor (for example, VEGF) is implanted in the cornea. VEGF diffuses from the pellet into the corneal tissue and stimulates endothelial cells lining existing vessels at the base to form sprouts. The sprouts then migrate toward the pellet along spatial gradients in VEGF. This example follows a common modeling paradigm where agent-based representations of cells are not included, but individual vessels are (2). As shown in Fig. 3 *b*, the cornea is represented as a hemispherical domain of radius 1.4 mm and thickness 0.1 mm, generated using the geometry component and meshed with linear tetrahedra using the Microvessel Chaste mesh component. The pellet is a cuboid with side length 0.3 mm and depth 0.1 mm, and a prescribed VEGF concentration of 3.0 nM on the boundaries. In practice, the VEGF in the pellet will deplete. Over time, vessels sprout from a large preexisting vessel at the base, or limbus, at a rate dependent on VEGF concentration. Then, they follow a persistent random walk, biased toward other vessels and positive VEGF gradients, as previously described. The VEGF distribution is obtained by solving the reaction-diffusion PDE in Eq. 1 on the cornea at the start of the simulation, in the absence of vessel or cell terms, with a fixed concentration maintained on the pellet by means of a Dirichlet boundary condition. For simplicity, the PDE solution is then fixed for the remainder of the simulation. In this case, a finite-element solver is used, available in the Chaste PDE component.

Vessels migrate toward the pellet, remaining within the volume of the 3D cornea geometry, with this boundary condition facilitated by tools in the geometry component. Possible extensions to this simple model include the addition of discrete stromal cells, distinction between perfused and unperfused vessels, subcellular signaling, VEGF depletion and consumption by cells, and the use of multiple vessel growth factors, as per Connor et al. (26).

## Conclusions

Microvessel Chaste, a library for composing multiphysical, multiscale spatial models of vascularized tissues, has been demonstrated, and two reproducible sample problems in the areas of tumor growth and angiogenesis presented. Familiarity with object-oriented programming, C++ or Python, and standard numerical methods are recommended. Users can develop their own tissue models, vascularized or otherwise, using a variety of discrete or spatially resolved representations of cells and blood vessels. Combination with existing Python packages for image processing and statistical analysis further enhances integration into experimental data processing workflows.

As shown in Fig. 1, the library can also be used to construct more general custom simulation software, for subsequent use by end-users less familiar with programming. GUIs can be created for specific tasks using wxPython, for example, and the 3D rendering back-end included in the library. Given these possibilities, we believe that the library will be useful for detailed model cross comparisons, closer integration of modeling and experimental data, and exploration of suitable coupling and time-stepping schemes for multiscale phenomena. All of these are major challenges in biophysical modeling.

There are some limitations. Windows and MacOS support are currently only available through Docker images, but work is ongoing to develop releases for these platforms. At present, most algorithms have not been parallelized, but all PDE and flow solvers are based on PETSc structures and the vessel network components may be communicated across processors using existing serialization functionality in Chaste (27). Additional functionality for semiautomated 2D and 3D image segmentation and meshing is under development. This includes the ability to generate finite-element meshes from 2D and 3D images automatically. It is envisaged that this will further aid integration with experimental studies such as those shown in Fig. 2 *a*.

The library is available under a permissive BSD license, with source files and documentation available via the project Github page https://jmsgrogan.github.io/MicrovesselChaste/. Contributions are welcome via Github pull requests and issues can be reported via the Github issue tracker. The latest release, version 3.4.2, is archived at http://dx.doi.org/10.5281/zenodo.213148.

## Author Contributions

J.A.G., A.J.C., P.K.M., H.M.B., and J.M.P.-F. designed the models and software. J.A.G., A.J.C., and J.M.P.-F. developed the software. B.M. and R.J.M. designed the experimental imaging. B.M. performed the experimental imaging. J.A.G., A.J.C., B.M., P.K.M., H.M.B., and J.M.P.-F. drafted and edited the article. All authors read and approved the final article.

## Supporting Material

Document S1. Supporting Materials and Methods

Document S2. Article plus Supporting Material
