# Supplementary material for: Microvessel Chaste: An Open Library for Spatial Modeling of Vascularized Tissues
Source: Biophys J. 2017 May 9;112(9):1767–72. doi: 10.1016/j.bpj.2017.03.036 (PMC5425404; doi:10.1016/j.bpj.2017.03.036)
Supplement: Document S1. Supporting Materials and Methods [file mmc1.pdf]

Biophysical Journal, Volume *112*

Supplemental Information

Microvessel Chaste: An Open Library for Spatial Modeling of Vascularized Tissues

James A. Grogan, Anthony J. Connor, Bostjan Markelc, Ruth J. Muschel, Philip K. Maini, Helen M. Byrne, and Joe M. Pitt-Francis

This tutorial is automatically generated from the file test/python/tutorials//TestPythonBiologicalNetworkLiteratePaper.py.

In [1]:
```
# Jupyter notebook specific imports
import matplotlib as mpl
from IPython import display
%matplotlib inline
```

A Tumour Growth Tutorial With A Real Network

This tutorial is designed to introduce a tumour growth problem based on a simplified version of the vascular tumour application described in Owen et al. 2011 (http://www.ncbi.nlm.nih.gov/pubmed/21363914).

It is a 3D simulation using cellular automaton for cells, lattice free migration for vessel movement and a regular grid for the solution of partial differential equations for oxygen and VEGF transport using the finite difference method.

The Test

In [2]:
```
import chaste # Core Chaste functionality
import chaste.cell_based # Chaste Cell Populations
chaste.init() # Initialize MPI and PETSc
import microvessel_chaste # Core Microvessel Chaste functionality
import microvessel_chaste.geometry # Geometry tools
import microvessel_chaste.mesh # Meshing
import microvessel_chaste.population.vessel # Vessel tools
import microvessel_chaste.pde # PDE and solvers
import microvessel_chaste.simulation # Flow and angiogenesis solvers
import microvessel_chaste.visualization # Visualization
from microvessel_chaste.utility import * # Dimensional analysis: bring in all units for convenience
# Set up the test
chaste.cell_based.SetupNotebookTest()
```

Set up output file management and seed the random number generator.

In [3]:
```
file_handler = chaste.core.OutputFileHandler("Python/TestBiologicalNetworkLiteratePaper")
chaste.core.RandomNumberGenerator.Instance().Reseed(12345)
```

This component uses explicit dimensions for all quantities, but interfaces with solvers which take non-dimensional inputs. The BaseUnits singleton takes time, length and mass reference scales to allow non-dimensionalisation when sending quantities to external solvers and re-dimensionalisation of results. For our purposes microns for length and hours for time are suitable base units.

In [4]:
```
reference_length = 1.e-6*metre()
reference_time = 3600.0*second()
reference_concentration = 1.e-6*mole_per_metre_cubed()
BaseUnits.Instance().SetReferenceLengthScale(reference_length)
BaseUnits.Instance().SetReferenceTimeScale(reference_time)
BaseUnits.Instance().SetReferenceConcentrationScale(reference_concentration)
```

Read a vessel network derived from biological images from file

In [5]:
```
vessel_reader = microvessel_chaste.population.vessel.VesselNetworkReader3()
vessel_reader.SetFileName("bio_original.vtp")
vessel_reader.SetMergeCoincidentPoints(True)
vessel_reader.SetTargetSegmentLength(40.0e-6*metre())
network = vessel_reader.Read()
```

The vessel network may contain short vessels due to image processing artifacts, we remove any vessels that are on the order of a single cell length and are not connected to other vessels at both ends. Note that units are explicitly specified for all quantities. It is ok to allow some small disconnected regions to remain for our purposes. The network is large, this can take up to 30 seconds.

In [6]:
```
short_vessel_cutoff = 40.0e-6 * metre()
remove_end_vessels_only = True
network.RemoveShortVessels(short_vessel_cutoff, remove_end_vessels_only)
network.UpdateAll()
network.MergeCoincidentNodes()
network.UpdateAll()
```

Write the modified network to file for inspection and visualize it.

In [7]:
```
network.Write(file_handler.GetOutputDirectoryFullPath() + "cleaned_network.vtp")
scene = microvessel_chaste.visualization.MicrovesselVtkScene3()
scene.SetVesselNetwork(network)
scene.GetVesselNetworkActorGenerator().SetEdgeSize(20.0)
nb_manager = microvessel_chaste.visualization.JupyterNotebookManager()
nb_manager.vtk_show(scene, height=600, width = 1000)
```

Out[7]:

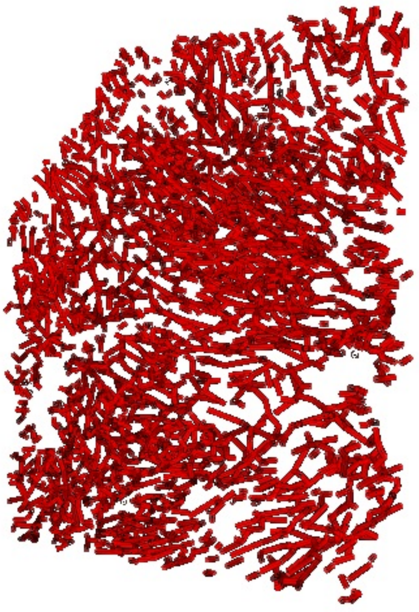

Simulating tumour growth for the entire network would be prohibitive for this tutorial, so we sample a small region. We can use some geometry tools to help.

In [8]:
```
cylinder = microvessel_chaste.geometry.Part3()
centre = microvessel_chaste.mesh.DimensionalChastePoint3(2300.0, 2300.0, -5.0, 1.e-6*metre())
radius = 600.0e-6*metre()
depth = 205.e-6*metre()
cylinder.AddCylinder(radius, depth, centre, 24)
cylinder.BooleanWithNetwork(network)
```

We visualize the smaller region

In [9]:
```
network.Write(file_handler.GetOutputDirectoryFullPath() + "cleaned_cut_network.vtp")
nb_manager.vtk_show(scene, height=600, width = 1000)
```

Out[9]:

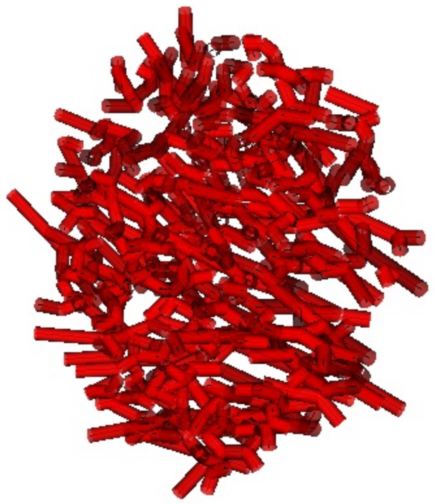

We are ready to simulate tumour growth and angiogenesis. We will use a regular lattice for this purpose. We size and position the lattice according to the bounds of the vessel network.

In [10]:
```
network_bounding_box = [microvessel_chaste.mesh.DimensionalChastePoint3(1500.0, 1600.0, -10.0, 1.e-6*metre()),
                        microvessel_chaste.mesh.DimensionalChastePoint3(3100.0, 3000.0, 300.0, 1.e-6*metre())]
grid = microvessel_chaste.mesh.RegularGrid3()
grid_spacing = 40.0e-6* metre()
grid.SetSpacing(grid_spacing)
```

We can use the built-in dimensional analysis functionality to get the network extents in terms of grid units

In [11]:
```
botom_front_left =  network_bounding_box[0].GetLocation(grid_spacing)
top_back_right =  network_bounding_box[1].GetLocation(grid_spacing)
extents = top_back_right - botom_front_left
extents = [int(x)+1 for x in extents] # snap to the nearest unit, overestimate size if needed
grid.SetExtents(extents)
network.Translate(microvessel_chaste.mesh.DimensionalChastePoint3(-1500.0, -1600.0, +10.0, 1.e-6*metre()))
```

Next we set the inflow and outflow boundary conditions for blood flow. Because the network connectivity is relatively low we assign all vessels near the top of the domain (z coord) as inflows and the bottom as outflows.

In [12]:
```
for eachNode in network.GetNodes():
    if eachNode.GetNumberOfSegments() == 1:
        if abs(eachNode.rGetLocation().GetLocation(1.e-6*metre())[2] -
               network_bounding_box[1].GetLocation(1.e-6*metre())[2]) < 80.0:
            eachNode.GetFlowProperties().SetIsInputNode(True)
            eachNode.GetFlowProperties().SetPressure(Owen11Parameters.mpInletPressure.GetValue("User"))
        elif abs(eachNode.rGetLocation().GetLocation(1.e-6*metre())[2] -
                 network_bounding_box[0].GetLocation(1.e-6*metre())[2]) < 80.0:
            eachNode.GetFlowProperties().SetIsOutputNode(True);
            eachNode.GetFlowProperties().SetPressure(Owen11Parameters.mpOutletPressure.GetValue("User"))
```

Again, we can write the network to file for visualization

In [13]:
```
network.Write(file_handler.GetOutputDirectoryFullPath() + "flow_boundary_labelled_network.vtp")
```

Next, set up the cell populations. We will setup up a population similar to that used in the Owen et al., 2011 paper. That is, a grid filled with normal cells and a tumour spheroid in the middle. We can use a generator for this purpose. The generator simply sets up the population using conventional Cell Based Chaste methods. It can take a few seconds to set up the population.

In [14]:
```
cell_population_genenerator = microvessel_chaste.population.cell.Owen11CellPopulationGenerator3()
cell_population_genenerator.SetRegularGrid(grid)
cell_population_genenerator.SetVesselNetwork(network)
tumour_radius = 300.0 * 1.e-6 * metre()
cell_population_genenerator.SetTumourRadius(tumour_radius)
cell_population = cell_population_genenerator.Update()
```

We can visualize the population. Note that we are reaching the limits of the browser based visualization at this point. The model can be better visualized in Paraview using the files we have been writing.

In [15]:
```
scene.SetCellPopulation(cell_population)
scene.GetCellPopulationActorGenerator().GetDiscreteColorTransferFunction().AddRGBPoint(1.0, 0.0, 0.0, 0.6)
scene.GetCellPopulationActorGenerator().SetPointSize(20)
scene.GetCellPopulationActorGenerator().SetColorByCellMutationState(True)
scene.ResetRenderer()
nb_manager.vtk_show(scene, height=600, width = 1000)
```

Out[15]:

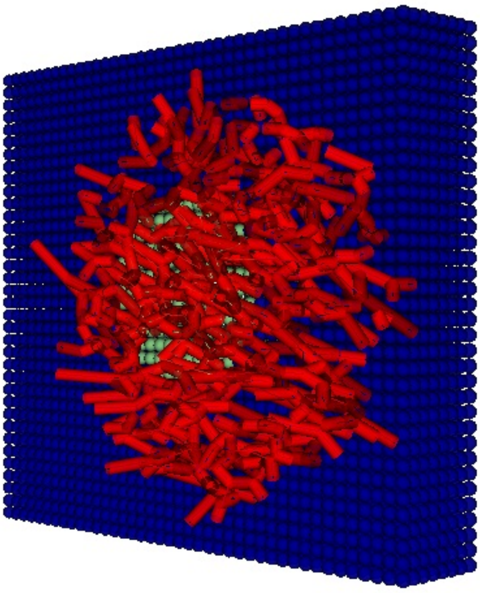

Next set up the PDEs for oxygen and VEGF. Cells will act as discrete oxygen sinks and discrete vegf sources.

In [16]:
```
oxygen_pde = microvessel_chaste.pde.LinearSteadyStateDiffusionReactionPde3_3()
oxygen_pde.SetIsotropicDiffusionConstant(Owen11Parameters.mpOxygenDiffusivity.GetValue("User"))
cell_oxygen_sink = microvessel_chaste.pde.CellBasedDiscreteSource3()
cell_oxygen_sink.SetLinearInUConsumptionRatePerCell(Owen11Parameters.mpCellOxygenConsumptionRate.GetValue("User"))
oxygen_pde.AddDiscreteSource(cell_oxygen_sink)
```

Vessels release oxygen depending on their haematocrit levels

In [17]:
```
vessel_oxygen_source = microvessel_chaste.pde.VesselBasedDiscreteSource3()
#oxygen_solubility_at_stp = Secomb04Parameters.mpOxygenVolumetricSolubility.GetValue("User") * GenericParameters.mpGasConce
ntrationAtStp.GetValue("User")
#vessel_oxygen_concentration = oxygen_solubility_at_stp * Owen11Parameters.mpReferencePartialPressure.GetValue("User")
vessel_oxygen_concentration = 0.02768 * mole_per_metre_cubed()
vessel_oxygen_source.SetReferenceConcentration(vessel_oxygen_concentration)
vessel_oxygen_source.SetVesselPermeability(Owen11Parameters.mpVesselOxygenPermeability.GetValue("User"))
vessel_oxygen_source.SetReferenceHaematocrit(Owen11Parameters.mpInflowHaematocrit.GetValue("User"))
oxygen_pde.AddDiscreteSource(vessel_oxygen_source);
```

Set up a finite difference solver and pass it the pde and grid.

In [18]:
```
oxygen_solver = microvessel_chaste.pde.FiniteDifferenceSolver3()
oxygen_solver.SetPde(oxygen_pde)
oxygen_solver.SetLabel("oxygen")
oxygen_solver.SetGrid(grid)
```

The rate of VEGF release depends on the cell type and intracellular VEGF levels, so we need a more detailed type of discrete source.

In [19]:
```
vegf_pde = microvessel_chaste.pde.LinearSteadyStateDiffusionReactionPde3_3()
vegf_pde.SetIsotropicDiffusionConstant(Owen11Parameters.mpVegfDiffusivity.GetValue("User"))
vegf_pde.SetContinuumLinearInUTerm(-1.0 * Owen11Parameters.mpVegfDecayRate.GetValue("User"))
```

Set up a map for different release rates depending on cell type. Also include a threshold intracellular VEGF below which there is no release.

In [20]:
```
normal_and_quiescent_cell_source = microvessel_chaste.pde.CellStateDependentDiscreteSource3()
normal_and_quiescent_cell_rates = microvessel_chaste.pde.MapUnsigned_ConcentrationFlowRate()
normal_and_quiescent_cell_rate_thresholds = microvessel_chaste.pde.MapUnsigned_Concentration()
quiescent_cancer_state = microvessel_chaste.population.cell.QuiescentCancerCellMutationState()
normal_cell_state = chaste.cell_based.WildTypeCellMutationState()
normal_and_quiescent_cell_rates[normal_cell_state.GetColour()] = Owen11Parameters.mpCellVegfSecretionRate.GetValue("User")
normal_and_quiescent_cell_rate_thresholds[normal_cell_state.GetColour()] = 0.27*mole_per_metre_cubed()
normal_and_quiescent_cell_rates[quiescent_cancer_state.GetColour()] = Owen11Parameters.mpCellVegfSecretionRate.GetValue("Use
r")
normal_and_quiescent_cell_rate_thresholds[quiescent_cancer_state.GetColour()] = 0.0*mole_per_metre_cubed()
normal_and_quiescent_cell_source.SetStateRateMap(normal_and_quiescent_cell_rates)
normal_and_quiescent_cell_source.SetLabelName("VEGF")
normal_and_quiescent_cell_source.SetStateRateThresholdMap(normal_and_quiescent_cell_rate_thresholds)
vegf_pde.AddDiscreteSource(normal_and_quiescent_cell_source)
```

Add a vessel related VEGF sink

In [21]:
```
vessel_vegf_sink = microvessel_chaste.pde.VesselBasedDiscreteSource3()
vessel_vegf_sink.SetReferenceConcentration(0.0*mole_per_metre_cubed())
vessel_vegf_sink.SetVesselPermeability(Owen11Parameters.mpVesselVegfPermeability.GetValue("User"))
vegf_pde.AddDiscreteSource(vessel_vegf_sink)
```

Set up a finite difference solver as before.

In [22]:
```
vegf_solver = microvessel_chaste.pde.FiniteDifferenceSolver3()
vegf_solver.SetPde(vegf_pde)
vegf_solver.SetLabel("VEGF_Extracellular")
vegf_solver.SetGrid(grid)
```

Next set up the flow problem. Assign a blood plasma viscosity to the vessels. The actual viscosity will depend on haematocrit and diameter. This solver manages growth and shrinkage of vessels in response to flow related stimuli.

In [23]:
```
large_vessel_radius = 25.0e-6 * metre()
network.SetSegmentRadii(large_vessel_radius)
viscosity = Owen11Parameters.mpPlasmaViscosity.GetValue("User")
network.SetSegmentViscosity(viscosity);
```

Set up the pre- and post flow calculators.

In [24]:
```
impedance_calculator = microvessel_chaste.simulation.VesselImpedanceCalculator3()
haematocrit_calculator = microvessel_chaste.simulation.ConstantHaematocritSolver3()
haematocrit_calculator.SetHaematocrit(Owen11Parameters.mpInflowHaematocrit.GetValue("User"))
wss_calculator = microvessel_chaste.simulation.WallShearStressCalculator3()
mech_stimulus_calculator = microvessel_chaste.simulation.MechanicalStimulusCalculator3()
metabolic_stim_calculator = microvessel_chaste.simulation.MetabolicStimulusCalculator3()
shrinking_stimulus_calculator = microvessel_chaste.simulation.ShrinkingStimulusCalculator3()
viscosity_calculator = microvessel_chaste.simulation.ViscosityCalculator3()
```

Set up and configure the structural adaptation solver.

In [25]:
```
structural_adaptation_solver = microvessel_chaste.simulation.StructuralAdaptationSolver3()
structural_adaptation_solver.SetTolerance(0.0001)
structural_adaptation_solver.SetMaxIterations(100)
structural_adaptation_solver.SetTimeIncrement(Owen11Parameters.mpVesselRadiusUpdateTimestep.GetValue("User"));
structural_adaptation_solver.AddPreFlowSolveCalculator(impedance_calculator)
structural_adaptation_solver.AddPostFlowSolveCalculator(haematocrit_calculator)
structural_adaptation_solver.AddPostFlowSolveCalculator(wss_calculator)
structural_adaptation_solver.AddPostFlowSolveCalculator(metabolic_stim_calculator)
structural_adaptation_solver.AddPostFlowSolveCalculator(mech_stimulus_calculator)
structural_adaptation_solver.AddPostFlowSolveCalculator(viscosity_calculator)
```

Set up a regression solver.

In [26]:
```
regression_solver = microvessel_chaste.simulation.WallShearStressBasedRegressionSolver3()
```

Set up an angiogenesis solver and add sprouting and migration rules.

In [27]:
```
angiogenesis_solver = microvessel_chaste.simulation.AngiogenesisSolver3()
sprouting_rule = microvessel_chaste.simulation.OffLatticeSproutingRule3()
sprouting_rule.SetSproutingProbability(1.e-5*per_second())
migration_rule = microvessel_chaste.simulation.OffLatticeMigrationRule3()
migration_rule.SetChemotacticStrength(0.1)
migration_rule.SetAttractionStrength(0.5)
migration_rule.SetSproutingVelocity((40.0*1.e-6/3600.0)*metre_per_second())
angiogenesis_solver.SetMigrationRule(migration_rule)
angiogenesis_solver.SetSproutingRule(sprouting_rule)
sprouting_rule.SetDiscreteContinuumSolver(vegf_solver)
migration_rule.SetDiscreteContinuumSolver(vegf_solver)
angiogenesis_solver.SetVesselNetwork(network)
```

The microvessel solver will manage all aspects of the vessel solve.

In [28]:
```
microvessel_solver = microvessel_chaste.simulation.MicrovesselSolver3()
microvessel_solver.SetVesselNetwork(network)
microvessel_solver.SetOutputFrequency(1)
microvessel_solver.AddDiscreteContinuumSolver(oxygen_solver)
microvessel_solver.AddDiscreteContinuumSolver(vegf_solver)
microvessel_solver.SetStructuralAdaptationSolver(structural_adaptation_solver)
microvessel_solver.SetRegressionSolver(regression_solver)
microvessel_solver.SetAngiogenesisSolver(angiogenesis_solver)
```

The microvessel solution modifier will link the vessel and cell solvers. We need to explicitly tell is which extracellular fields to update based on PDE solutions.

In [29]:
```
microvessel_modifier = microvessel_chaste.simulation.MicrovesselSimulationModifier3()
microvessel_modifier.SetMicrovesselSolver(microvessel_solver)
update_labels = microvessel_chaste.simulation.VecString()
update_labels.append("oxygen")
update_labels.append("VEGF_Extracellular")
microvessel_modifier.SetCellDataUpdateLabels(update_labels)
```

Set up plotting

In [30]:
```
scene.GetCellPopulationActorGenerator().SetColorByCellData(True)
scene.GetCellPopulationActorGenerator().SetDataLabel("oxygen")
scene_modifier = microvessel_chaste.visualization.JupyterMicrovesselSceneModifier3(nb_manager)
scene_modifier.SetVtkScene(scene)
scene_modifier.SetUpdateFrequency(1)
microvessel_solver.AddMicrovesselModifier(scene_modifier)
```

The full simulation is run as a typical Cell Based Chaste simulation

In [31]:
```
simulator = chaste.cell_based.OnLatticeSimulation3(cell_population)
simulator.AddSimulationModifier(microvessel_modifier)
```

Add a killer to remove apoptotic cells

In [32]:
```
apoptotic_cell_killer = chaste.cell_based.ApoptoticCellKiller3(cell_population)
simulator.AddCellKiller(apoptotic_cell_killer)
```

Add another modifier for updating cell cycle quantities.

In [33]:
```
owen11_tracking_modifier = microvessel_chaste.simulation.Owen2011TrackingModifier3()
simulator.AddSimulationModifier(owen11_tracking_modifier)
```

Set up the remainder of the simulation

In [34]:
```
simulator.SetOutputDirectory("Python/TestBiologicalNetworkLiteratePaper")
simulator.SetSamplingTimestepMultiple(1)
simulator.SetDt(0.5)
```

This end time corresponds to roughly 10 minutes run-time on a desktop PC. Increase it or decrease as preferred. The end time used in Owen et al. 2011 is 4800 hours.

In [35]:
```
simulator.SetEndTime(2.0)
```

Do the solve. A sample solution is shown at the top of this test.

In [36]:
```
simulator.Solve()
```

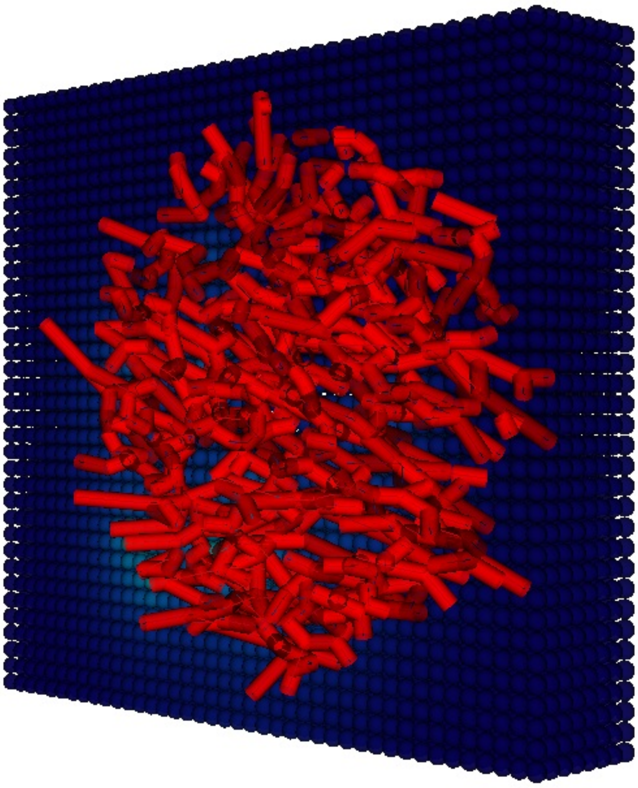

Dump the parameters to file for inspection.

In [37]:
```
ParameterCollection.Instance().DumpToFile(file_handler.GetOutputDirectoryFullPath()+"parameter_collection.xml")
nb_manager.add_parameter_table(file_handler)
# Tear down the test
chaste.cell_based.TearDownNotebookTest()
```

name	value	symbol	added_by	descrip
				Gas

Generic_GasConcentrationAtStp	44.6429 m^-3 mol	C_{stp}	Owen2011OxygenBasedCellCycleOdeSystem	concent at STP
Owen11_BasalMetabolicStimulus	1.7 Hz	k_m^0	MetabolicStimulusCalculator	Basal metabol stimulus
Owen11_CellMotilityCancer	8.33333e-15 m^2 s^-1	D_{cancer}	Owen11CaUpdateRule	Maximu cell mot cancer
Owen11_CellOxygenConsumptionRate	0.216667 Hz	k_c^{cell}	User	Cell oxy consum rate
Owen11_CellVegfProductionRate	3.33333e-05 Hz	k_8	Owen2011OxygenBasedCellCycleOdeSystem	Basal V producti rate in c
Owen11_CellVegfSecretionRate	1.66667e-13 m^-3 s^-1 mol	k_v^{cell}	User	Cell veg secretio rate
Owen11_CriticalWallShearStress	0.8 Pa	τ_{wall}	WallShearStressBasedRegressionSolver	Critical shear st for vess pruning
Owen11_InflowHaematocrit	0.45 dimensionless	H_{in}	User	Inflow haemat
Owen11_InletPressure	3333.05 Pa	P_{in}	User	Vessel network pressur
Owen11_MaxCellVegfProductionRate	0.000166667 Hz	k_{8*}	Owen2011OxygenBasedCellCycleOdeSystem	Max VE producti rate in c
Owen11_MaxTimeWithLowWallShearStress	240000 s	T_{prune}	WallShearStressBasedRegressionSolver	Maximu vessel survivia with low shear st
Owen11_MaximumRadius	5e-05 m	R_{MAX}	RadiusCalculator	Maximu possible radius
Owen11_MaximumSproutingRate	4.16667e-06 Hz	P_{sprout}^{max}	Owen2011SproutingRule	Maximu rate of sproutin
Owen11_MinCellCycleCancer	96000 s	T_{min}^{cancer}	Owen2011OxygenBasedCellCycleOdeSystem	Minimur cycle pe cancer
Owen11_MinCellCycleNormal	180000 s	T_{min}^{normal}	Owen2011OxygenBasedCellCycleOdeSystem	Minimur cycle pe normal
Owen11_MinimumRadius	1e-06 m	R_{MIN}	RadiusCalculator	Minimur possible radius
Owen11_OutletPressure	1999.83 Pa	P_{out}	User	Vessel network outlet pressur
				Oxygen

Owen11_OxygenAtHalfMaxCycleRateCancer	186.651 Pa	C^{cancer}	Owen2011OxygenBasedCellCycleOdeSystem	partial pressur[illegible] half ma[illegible] cycle ra[illegible] cancer
Owen11_OxygenAtHalfMaxCycleRateNormal	399.966 Pa	C^{normal}	Owen2011OxygenBasedCellCycleOdeSystem	Oxygen partial pressur[illegible] half ma[illegible] cycle ra[illegible] normal
Owen11_OxygenAtQuiescence	1186.57 Pa	C^{enter}_{quiesc}	Owen2011OxygenBasedCellCycleModel	Oxygen partial pressur[illegible] quiesce[illegible]
Owen11_OxygenDiffusivity	2.41667e-09 m^2 s^-1	D_c	User	Oxygen diffusivi[illegible]
Owen11_OxygenLeaveQuiescence	1306.56 Pa	C^{leave}_{quiesc}	Owen2011OxygenBasedCellCycleModel	Oxygen partial pressur[illegible] leave quiesce[illegible]
Owen11_OxygenTensionForHalfMaxP53Degradation	591.95 Pa	C_{p53}	Owen2011OxygenBasedCellCycleOdeSystem	Tissue oxygen tension half-ma[illegible] degrada[illegible]
Owen11_OxygenTensionForHalfMaxVegfDegradation	591.95 Pa	C_{VEGF}	Owen2011OxygenBasedCellCycleOdeSystem	Tissue oxygen tension half-ma[illegible] degrada[illegible]
Owen11_P53EffectOnVegfProduction	-3.33333e-05 Hz	k_{8**}	Owen2011OxygenBasedCellCycleOdeSystem	Effect o[illegible] on VEG[illegible] producti[illegible]
Owen11_P53MaxDegradationRate	0.000166667 Hz	k_{*7}	Owen2011OxygenBasedCellCycleOdeSystem	Max p5[illegible] degrada[illegible] rate
Owen11_P53ProductionRateConstant	3.33333e-05 Hz	k_7	Owen2011OxygenBasedCellCycleOdeSystem	Intracell[illegible] p53 producti[illegible] rate cor[illegible]
Owen11_PlasmaViscosity	0.0012 m^-1 kg s^-1	μ_{plasma}	ViscosityCalculator	Blood plasma viscosity[illegible]
Owen11_ReferenceFlowRateForMetabolicStimulus	6.66667e-13 m^3 s^-1	Q_{ref}	MetabolicStimulusCalculator	Referen[illegible] flow rate[illegible] metabol[illegible] stimulus[illegible]
Owen11_SensitivityToIntravascularPressure	0.5 Hz	k_p	MechanicalStimulusCalculator	Shrinkir[illegible] intravas[illegible] pressur[illegible]
Owen11_ShrinkingTendency	1.7 Hz	k_s	ShrinkingStimulusCalculator	Shrinkir[illegible] tendenc[illegible]
Owen11_TimeDeathQuiescence	240000 s	T_{death}	Owen2011OxygenBasedCellCycleModel	Time for[illegible] death d[illegible] sustaine[illegible]

				quiesce
Owen11_VegfConventrationAtHalfMaxProbSprouting	5e-10 m^-3 mol	V_{sprout}	Owen2011SproutingRule	VEGF concent at half maxima vessel sproutin probabil
Owen11_VegfDecayRate	0.000166667 Hz	δ_v	User	Vegf de rate
Owen11_VegfDiffusivity	1.66667e-11 m^2 s^-1	D_v	User	Vegf diffusivi
Owen11_VegfEffectOnVegfProduction	0.04 dimensionless	j_5	Owen2011OxygenBasedCellCycleOdeSystem	Effect o VEGF o VEGF producti
Owen11_VesselOxygenPermeability	0.001 m s^-1	ψ_c	User	Vessel permea to oxyge
Owen11_VesselRadiusUpdateTimestep	0.1 s	t	User	Vessel r update timestep
Owen11_VesselVegfPermeability	1.66667e-09 m s^-1	ψ_v	User	Vessel permea to vegf
Secomb04_OxygenVolumetricSolubility	2.3252e-07 m kg^-1 s^2	α_{eff}	Owen2011OxygenBasedCellCycleOdeSystem	Oxygen solubility

In [ ]:

This tutorial is automatically generated from the file test/python/tutorials//TestPythonOffLatticeAngiogenesisLiteratePaper.py.

In [1]:
```
# Jupyter notebook specific imports
import matplotlib as mpl
from IPython import display
%matplotlib inline
```

An Off Lattice Angiogenesis Tutorial

This tutorial demonstrates functionality for modelling 3D off-lattice angiogenesis in a corneal micro pocket application, similar to that described in Connor et al. 2015 (http://rsif.royalsocietypublishing.org/content/12/110/20150546.abstract).

It is a 3D simulation modelling VEGF diffusion and decay from an implanted pellet using finite element methods and lattice-free angiogenesis from a large limbal vessel towards the pellet.

The Test

In [2]:
```
import numpy as np
import chaste # Core Chaste functionality
import chaste.cell_based # Chaste Cell Populations
chaste.init() # Initialize MPI and PETSc
import microvessel_chaste # Core Microvessel Chaste functionality
import microvessel_chaste.geometry # Geometry tools
import microvessel_chaste.mesh # Meshing
import microvessel_chaste.population.vessel # Vessel tools
import microvessel_chaste.pde # PDE and solvers
import microvessel_chaste.simulation # Flow and angiogenesis solvers
import microvessel_chaste.visualization # Visualization
from microvessel_chaste.utility import * # Dimensional analysis: bring in all units for convenience
# Set up the test
chaste.cell_based.SetupNotebookTest()
```

Set up output file management.

In [3]:
```
file_handler = chaste.core.OutputFileHandler("Python/TestOffLatticeAngiogenesisLiteratePaper")
chaste.core.RandomNumberGenerator.Instance().Reseed(12345)
```

This component uses explicit dimensions for all quantities, but interfaces with solvers which take non-dimensional inputs. The BaseUnits singleton takes time, length and mass reference scales to allow non-dimensionalisation when sending quantities to external solvers and re-dimensionalisation of results. For our purposes microns for length and hours for time are suitable base units.

In [4]:
```
reference_length = 1.e-6 * metre()
reference_time = 3600.0 * second()
reference_concentration = 1.e-9*mole_per_metre_cubed()
BaseUnits.Instance().SetReferenceLengthScale(reference_length)
BaseUnits.Instance().SetReferenceTimeScale(reference_time)
BaseUnits.Instance().SetReferenceConcentrationScale(reference_concentration)
```

Set up the domain representing the cornea. This is a thin hemispherical shell. We assume some symmetry to reduce computational expense.

In [5]:
```
hemisphere_generator = microvessel_chaste.geometry.MappableGridGenerator()
radius = 1400.0e-6*metre()
thickness = 100.0e-6*metre()
num_divisions_x = 10
num_divisions_y = 10
azimuth_angle = 1.0 * np.pi
polar_angle = 0.5 * np.pi
cornea = hemisphere_generator.GenerateHemisphere(radius/reference_length,
                                                 thickness/reference_length,
                                                 num_divisions_x,
                                                 num_divisions_y,
                                                 azimuth_angle,
                                                 polar_angle)
```

We can visualize the part

In [6]:
```
scene = microvessel_chaste.visualization.MicrovesselVtkScene3()
scene.SetPart(cornea)
scene.GetPartActorGenerator().SetVolumeOpacity(0.7)
scene.GetPartActorGenerator().SetVolumeColor((255.0, 255.0, 255.0))
nb_manager = microvessel_chaste.visualization.JupyterNotebookManager()
nb_manager.vtk_show(scene, height=600, width = 1000)
```

Out[6]:

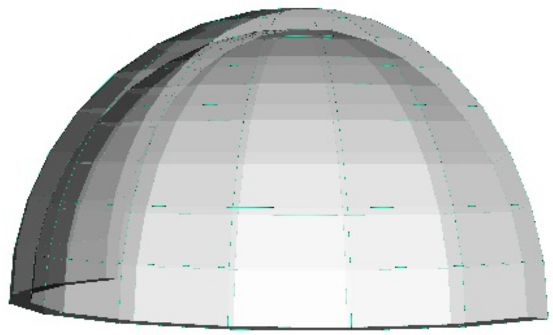

Set up a vessel network, with divisions roughly every 'cell length'. Initially it is straight. We will map it onto the hemisphere.

In [7]:
```
network_generator = microvessel_chaste.population.vessel.VesselNetworkGenerator3()
vessel_length = np.pi * radius
cell_length = 40.0e-6 * metre()
origin = microvessel_chaste.mesh.DimensionalChastePoint3(0.0, 4000.0, 0.0)
network  = network_generator.GenerateSingleVessel(vessel_length, origin, int(float(vessel_length/cell_length)) + 1, 0)
network.GetNode(0).GetFlowProperties().SetIsInputNode(True);
network.GetNode(0).GetFlowProperties().SetPressure(Owen11Parameters.mpInletPressure.GetValue("User"))
network.GetNode(network.GetNumberOfNodes()-1).GetFlowProperties().SetIsOutputNode(True)
network.GetNode(network.GetNumberOfNodes()-1).GetFlowProperties().SetPressure(Owen11Parameters.mpOutletPressure.GetValue(
"User"))
nodes = network.GetNodes();
for eachNode in nodes:
    node_azimuth_angle = float(azimuth_angle * eachNode.rGetLocation().GetLocation(reference_length)[0]*reference_length/vessel_len
gth)
    node_polar_angle = float(polar_angle*eachNode.rGetLocation().GetLocation(reference_length)[1]*reference_length/vessel_length)
    dimless_radius = (float(radius/reference_length)+(-0.5*float(thickness/reference_length)))
    new_position = microvessel_chaste.mesh.DimensionalChastePoint3(dimless_radius * np.cos(node_azimuth_angle) * np.sin(node_pol
ar_angle),
                                                                   dimless_radius * np.cos(node_polar_angle),
                                                                   dimless_radius * np.sin(node_azimuth_angle) * np.sin(node_polar_angle),
                                                                   reference_length)
    eachNode.SetLocation(new_position)
```

Visualize the network

In [8]:
```
scene.SetVesselNetwork(network)
scene.GetVesselNetworkActorGenerator().SetEdgeSize(20.0)
nb_manager.vtk_show(scene, height=600, width = 1000)
```

Out[8]:

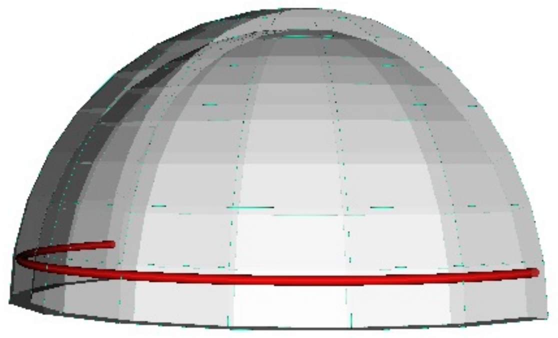

In the experimental assay a pellet containing VEGF is implanted near the top of the cornea. We model this as a fixed concentration of VEGF in a cuboidal region. First set up the vegf sub domain.

In [9]:
```
pellet = microvessel_chaste.geometry.Part3()
pellet_side_length = 300.0e-6 * metre()
origin = microvessel_chaste.mesh.DimensionalChastePoint3(-150.0,900.0,0.0)
pellet.AddCuboid(pellet_side_length, pellet_side_length, 5.0*pellet_side_length, origin)
pellet.Write(file_handler.GetOutputDirectoryFullPath()+"initial_vegf_pellet.vtp",
          microvessel_chaste.geometry.GeometryFormat.VTP)
```

Now make a finite element mesh on the cornea.

In [10]:
```
mesh_generator = microvessel_chaste.mesh.DiscreteContinuumMeshGenerator3_3()
mesh_generator.SetDomain(cornea)
mesh_generator.SetMaxElementArea(1e-6 * metre_cubed())
mesh_generator.Update()
mesh = mesh_generator.GetMesh()
```

We can visualize the mesh

In [11]:
```
scene.GetPartActorGenerator().SetVolumeOpacity(0.0)
scene.SetMesh(mesh)
nb_manager.vtk_show(scene, height=600, width = 1000)
```

Out[11]:

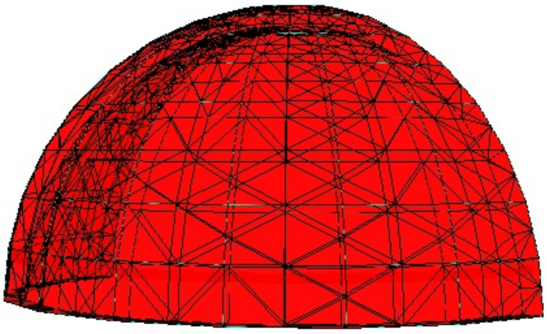

Set up the vegf pde. Note the scaling of the refernece concentration to nM to avoid numerical precision problems.

In [12]:
```
vegf_pde = microvessel_chaste.pde.LinearSteadyStateDiffusionReactionPde3_3()
vegf_pde.SetIsotropicDiffusionConstant(Owen11Parameters.mpVegfDiffusivity.GetValue("User"))
vegf_pde.SetContinuumLinearInUTerm(-1.0*Owen11Parameters.mpVegfDecayRate.GetValue("User"))
vegf_pde.SetMesh(mesh)
vegf_pde.SetUseRegularGrid(False)
vegf_pde.SetReferenceConcentration(1.e-9*mole_per_metre_cubed())
```

Add a boundary condition to fix the VEGF concentration in the vegf subdomain.

In [13]:
```
vegf_boundary = microvessel_chaste.pde.DiscreteContinuumBoundaryCondition3()
vegf_boundary.SetType(microvessel_chaste.pde.BoundaryConditionType.IN_PART)
vegf_boundary.SetSource(microvessel_chaste.pde.BoundaryConditionSource.PRESCRIBED)
vegf_boundary.SetValue(3.e-9*mole_per_metre_cubed())
vegf_boundary.SetDomain(pellet)
```

Set up the PDE solvers for the vegf problem.

In [14]:
```
vegf_solver = microvessel_chaste.pde.FiniteElementSolver3()
vegf_solver.SetPde(vegf_pde)
vegf_solver.SetLabel("vegf")
vegf_solver.SetMesh(mesh)
vegf_solver.AddBoundaryCondition(vegf_boundary)
```

Set up an angiogenesis solver and add sprouting and migration rules.

In [15]:
```
angiogenesis_solver = microvessel_chaste.simulation.AngiogenesisSolver3()
sprouting_rule = microvessel_chaste.simulation.OffLatticeSproutingRule3()
sprouting_rule.SetSproutingProbability(1.e6* per_second())
migration_rule = microvessel_chaste.simulation.OffLatticeMigrationRule3()
migration_rule.SetChemotacticStrength(0.1)
migration_rule.SetAttractionStrength(0.5)
sprout_velocity = (50.0e-6/(24.0*3600.0))*metre_per_second() #Secomb13
migration_rule.SetSproutingVelocity(sprout_velocity)
angiogenesis_solver.SetMigrationRule(migration_rule)
angiogenesis_solver.SetSproutingRule(sprouting_rule)
sprouting_rule.SetDiscreteContinuumSolver(vegf_solver)
migration_rule.SetDiscreteContinuumSolver(vegf_solver)
angiogenesis_solver.SetVesselNetwork(network)
angiogenesis_solver.SetBoundingDomain(cornea)
```

Set up the MicrovesselSolver which coordinates all solves.

In [16]:
```
microvessel_solver = microvessel_chaste.simulation.MicrovesselSolver3()
microvessel_solver.SetVesselNetwork(network)
microvessel_solver.AddDiscreteContinuumSolver(vegf_solver)
microvessel_solver.SetOutputFileHandler(file_handler)
microvessel_solver.SetOutputFrequency(5)
microvessel_solver.SetAngiogenesisSolver(angiogenesis_solver)
microvessel_solver.SetUpdatePdeEachSolve(False)
```

Set up plotting

In [17]:
```
scene.GetDiscreteContinuumMeshActorGenerator().SetVolumeOpacity(0.3)
scene.GetDiscreteContinuumMeshActorGenerator().SetDataLabel("Nodal Values")
scene.GetVesselNetworkActorGenerator().SetEdgeSize(5.0)
scene_modifier = microvessel_chaste.visualization.JupyterMicrovesselSceneModifier3(nb_manager)
scene_modifier.SetVtkScene(scene)
scene_modifier.SetUpdateFrequency(2)
microvessel_solver.AddMicrovesselModifier(scene_modifier)
```

Set the simulation time and run the solver.

In [18]:
```
chaste.cell_based.SimulationTime.Instance().SetEndTimeAndNumberOfTimeSteps(100.0, 10)
microvessel_solver.Run()
```

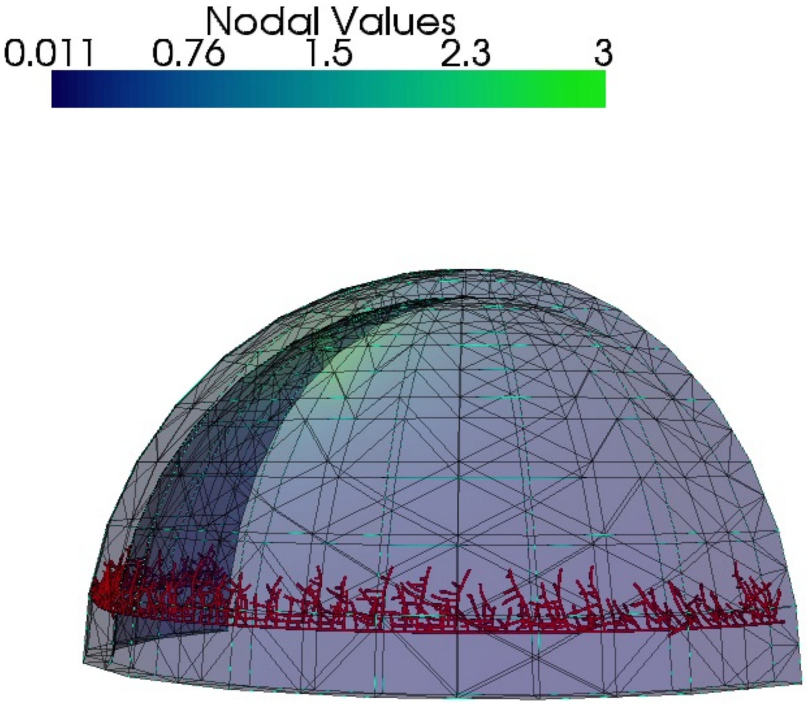

Dump the parameters to file for inspection.

In [19]:
```
ParameterCollection.Instance().DumpToFile(file_handler.GetOutputDirectoryFullPath()+"parameter_collection.xml")
nb_manager.add_parameter_table(file_handler)
# Tear down the test
chaste.cell_based.TearDownNotebookTest()
```

name	value	symbol	added_by	description
Owen11_InletPressure	3333.05 Pa	P_{in}	User	Vessel network inlet pressure$
Owen11_MaximumSproutingRate	4.16667e-06 Hz	P^{max}_{sprout}	Owen2011SproutingRule	Maximum rate of sprouting
Owen11_OutletPressure	1999.83 Pa	P_{out}	User	Vessel network outlet pressure
Owen11_VegfConventrationAtHalfMaxProbSprouting	5e-10 m^-3 mol	V_{sprout}	Owen2011SproutingRule	VEGF concentration at half maximal vessel sprouting probability
Owen11_VegfDecayRate	0.000166667 Hz	δ_v	User	Vegf decay rate
Owen11_VegfDiffusivity	1.66667e-11 m^2 s^-1	D_v	User	Vegf diffusivity

In [ ]:
